# CT and DSA for evaluation of spontaneous intracerebral lobar bleedings

**DOI:** 10.3389/fneur.2022.956888

**Published:** 2022-10-03

**Authors:** Jens-Christian Altenbernd, Sebastian Fischer, Wolfram Scharbrodt, Sebastian Schimrigk, Jens Eyding, Hannes Nordmeyer, Christine Wohlert, Nils Dörner, Yan Li, Karsten Wrede, Daniela Pierscianek, Martin Köhrmann, Benedikt Frank, Michael Forsting, Cornelius Deuschl

**Affiliations:** ^1^Department of Radiology, Gemeinschaftskrankenhaus, Herdecke, Germany; ^2^Institute of Diagnostic and Interventional Radiology and Neuroradiology, University Hospital Essen, Essen, Germany; ^3^Department of Radiology, Knappschaftskrankenhaus, Bochum, Germany; ^4^Department of Neurosurgery, Gemeinschaftskrankenhaus, Herdecke, Germany; ^5^Department of Neurology, Gemeinschaftskrankenhaus, Herdecke, Germany; ^6^Radprax, Solingen, Germany; ^7^Department of Neurosurgery, University Hospital Essen, Essen, Germany; ^8^Department of Neurology, University Hospital Essen, Essen, Germany

**Keywords:** atypical bleeding, ICH, CT, DSA, diagnostic

## Abstract

**Purpose:**

This study retrospectively examined the extent to which computed tomography angiography (CTA) and digital subtraction angiography (DSA) can help identify the cause of lobar intracerebral bleeding.

**Materials and methods:**

In the period from 2002 to 2020, data from patients who were >18 years at a university and an academic teaching hospital with lobar intracerebral bleeding were evaluated retrospectively. The CTA DSA data were reviewed separately by two neuroradiologists, and differences in opinion were resolved by consensus after discussion. A positive finding was defined as an underlying vascular etiology of lobar bleeding.

**Results:**

The data of 412 patients were retrospectively investigated. DSA detected a macrovascular cause of bleeding in 125/412 patients (33%). In total, sixty patients had AVMs (15%), 30 patients with aneurysms (7%), 12 patients with vasculitis (3%), and 23 patients with dural fistulas (6%). The sensitivity, specificity, positive and negative predictive values, and accuracy of CTA compared with DSA were 93, 97, 100, and 97%. There were false-negative CTA readings for two AVMs and one dural fistula.

**Conclusion:**

The DSA is still the gold standard diagnostic modality for detecting macrovascular causes of ICH; however, most patients with lobar ICH can be investigated first with CTA, and the cause of bleeding can be found. Our results showed higher sensitivity and specificity than those of other CTA studies.

## Introduction

Intracerebral hemorrhage (ICH) results from the rupture of blood vessels within the brain parenchyma. The ruptured vessel is usually abnormally formed, like in the case of a vascular malformation, or has been damaged by a chronic or acute pathologic process. ICH occurs as a complication of several diseases, the most prevalent of which is chronic hypertension. ICH is the most common type of hemorrhagic stroke, accounting for ~10% of all strokes with an incidence of between 7 and 17 per 100,000 (1). Despite aggressive management strategies, the 30-day mortality remains high, at up to 50%, with the majority of deaths occurring in the first 2 days (2–4). At 6 months, only 20–30% achieve independent status (5, 6). The remaining survivors place a significant burden on the healthcare system (1–4). Intracerebral hemorrhage (ICH) can be divided into central and lobar ICH. Central ICH occurs in the basal ganglia, thalamus, brain stem, and mainly presents with a hypertensive etiology. Most of the other bleeding events are called lobar, mostly irregular shaped or with intraventricular parts, and are therefore not a result of increased blood pressure (3, 5, 6). Deep ICH has been attributed to long-standing hypertensive arteriopathy. Acute management as well as establishing the etiology of an intracerebral hemorrhage is still a challenge for clinicians. The location of the intracerebral hemorrhage alone should not be used to determine the cause because atypically located hemorrhages can be caused by long-standing arterial hypertension and typically located hemorrhages can occur due to non-hypertensive causes (7, 8) (Table 1).

**Table 1:** Baseline characteristics.

**Baseline characteristics**	**All ICH patients**	**Macrovascular cause**	**Non-macrovasular cause**
	***N* = 412**	***N* =113**	***N* =299**
Sex	62% male	66% male	62% male
Age	53 y ± 15	42 y ± 12	56 y ± 14
Hypertension	28%	22%	32%
Small vessel disease	35%	7%	52%
Posterior fossa	14%	24%	8%
Antigoagulation	16%	12%	19%
NIHSS	19 ± 6	17 ± 5	21 ± 4
GCS	9 ± 4	8 ± 4	10 ± 5
Median time (h) between CTA and DSA	6.0 ± 2.3	6.3 ± 2.1	5.9 ± 2.4

Accurately distinguishing non-traumatic intracerebral hemorrhage (ICH) subtypes is important since they may have different risk factors, causal pathways, management, and prognosis. The reliability of existing classification systems appears excellent but is unknown outside specialist centers with experienced raters (9).

Computed tomography (CT), digital subtraction angiography (DSA), and magnetic resonance imaging/angiography (MRI/MRA) are equivalent for the detection of acute bleeding (10). Owing to the availability and speed of the examination, CT is preferred, with the exception of pregnant and young patients (11). If cross-sectional imaging suggests that vascular malformation is the cause of bleeding, DSA is indicated as a further diagnostic method and is considered to be the gold standard for diagnosing cerebrovascular pathologies (12).

The present study retrospectively examined the extent to which computed tomography angiography (CTA) can identify the vascular cause of lobar intracerebral bleeding compared to DSA.

## Materials and methods

**Figure 1:**
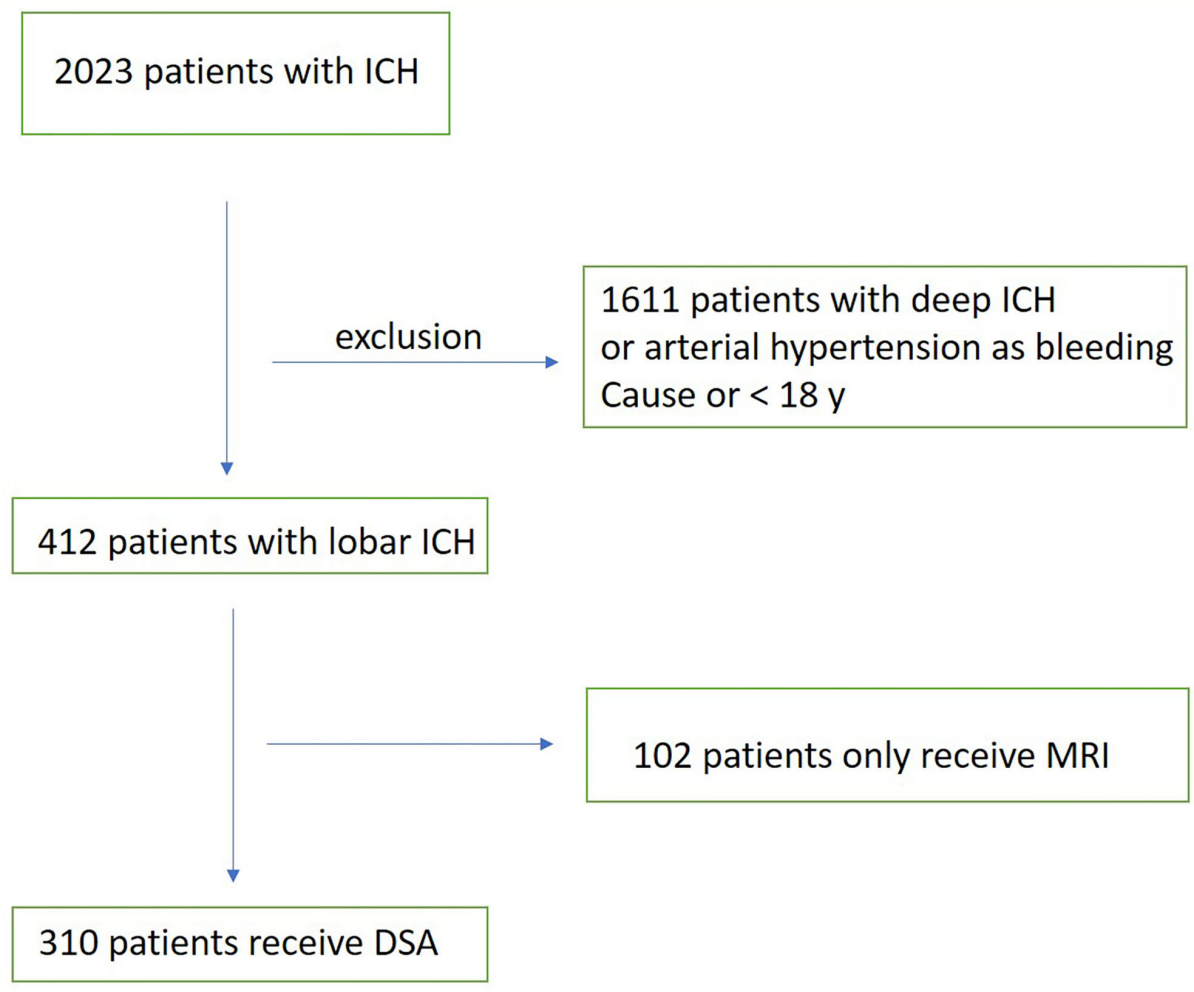
Inclusion flow chart.

Within the period from 2002 to 2020, the data from patients at a university and an academic teaching hospital with lobar intracerebral bleeding were evaluated retrospectively (see inclusion flow chart Figure 1). As a standard examination all included patients were investigated using non-contrast CT, CTA, and DSA; 85% of these patients were also investigated using MRI/MRA. Patients were >18 years. Patients who experienced bleeding with increased intracranial pressure as the cause of emergency craniotomy before DSA were excluded from the study.

Axial images of non-contrast CT and CTA DSA data were all reviewed separately by two neuroradiologists, and differences in assessments were resolved by consensus after discussion. A positive finding was defined as the underlying vascular etiology of the lobular bleeding.

The CT angiography was performed using a multidetector CT scanner with 128 and 320 slices. Unenhanced brain CT with a 5-mm maximum slice thickness was performed, and CT angiography was performed.

The DSA consisted of selective catheterization of the internal and external carotid arteries and vertebral arteries with biplanar angiography systems under general anesthesia (Siemens, Canon).

The MRI/MRA studies were done on 1.5T or 3T magnetic resonance scanners and included a transversal T1-weighted scan, T2 turbo-spin-echo, and T2 FLAIR, 3D time-of-flight MRI/MRA after gadolinium contrast injection and a transversal 3D T1-weighted contrast enhanced scan.

The study was performed according to the Standards for Reporting of Diagnostic.

Accuracy (STARD) (13) and was approved by the local ethics committee.

Descriptive statistics included the number of observations, mean and standard deviation (SD), median and interquartile range for continuous variables, and counts and percentages for discrete variables. Statistical analysis was performed using SAS v 9.4 (SAS Institute, Cary, North Carolina, USA).

## Results

Between 2002 and 2020, 2,023 patients with ICH were investigated. In total, data of 412 patients were retrospectively included with lobar bleeding, and no arterial hypertension was recorded as a cause of bleeding, the mean age of participants was 53.4 (±14.9) years, and 276 (67%) were male. The median interval between CTA and DSA was 6 h (±2.3 h). All the patients received CT, CTA, and DSA, of which 85% were also investigated with MRI/MRA.

Baseline characteristics are shown in Table 1.

**Figure 2:**
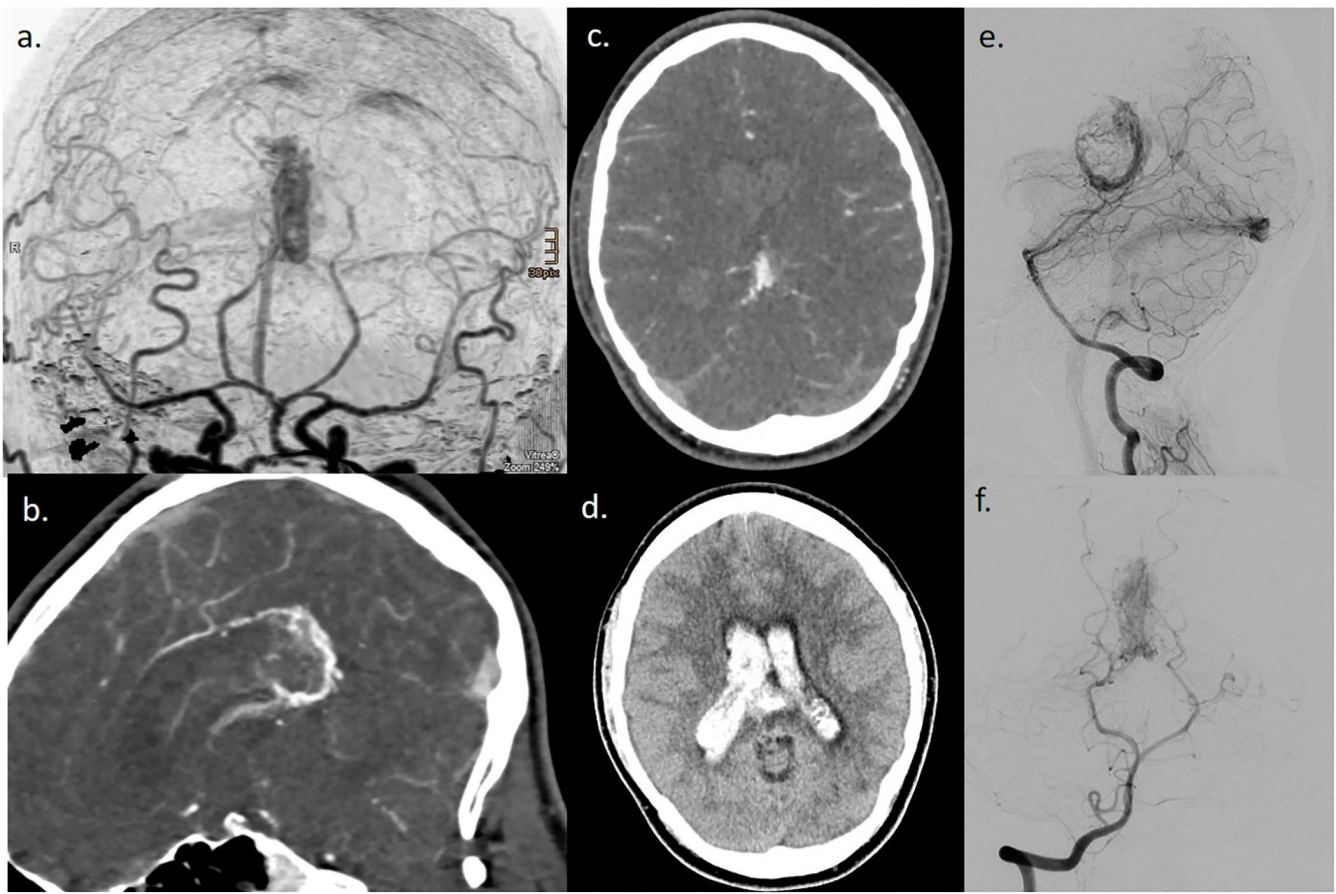
A case of a 28-year-old man with lobar ICH, and CT DSA images revealed no arterial hypertension. **(a–d)** CT/CTA images; **(e,f)** DSA images of an AVM at the splenium with hemorrhage.

The DSA detected a macrovascular cause of bleeding in 125/412 patients (33%). Out of the 125 patients, 60 patients had AVMs (15%) (Figure 2), 30 patients had aneurysms (7%), 12 patients had vasculitis (3%), and 23 patients had dural arterio-venous fistulas (dural AV fistula) (6%) (Table 2).

**Table 2:** Bleeding causes.

**Underlying causes for non-hypertensive lobar intracerebral hemorrhage**	**Number of patients**
Aneurysm	30/412
Arteriovenous malformation	60/412
Dural AV fistula	23/412
Vasculitis	12/412

In total, five DSA-negative cases were found to be related to tumors on MRI. The positive predictive value of MRI was 35% and the negative predictive value was 92%.

The sensitivity, specificity, positive, and negative predictive values of CTA compared with DSA were 97, 93, 100, and 97%, respectively. There were false-negative CTA readings for 2 AVMs and one dural fistula.

Cohen's Kappa for interobserver agreement of the two independent neuroradiologists was 0.85.

## Discussion

In our study 3/125 patients (2%) had false-negative CTA readings, where 2 AVMs < 2 cm and one small dural fistula were found only using DSA. The vascular pathologies missed by CTA were very small and were localized to the parietal lobar. The initial hematomas were poorly defined. Wu et al. reported 100% specificity and sensitivity of CTA in detecting AVMs larger than 2.0 cm with 100% accuracy for identifying arterial feeders and 82.6% accuracy for venous drainage (15).

According to the DIAGRAM Score in our lobar ICH patients younger age, lobar or posterior fossa location of ICH, absence of signs of small vessel disease, and a positive or inconclusive CTA were associated with a macrovascular cause (14).

A potential CTA limitation is the diagnosis of dAVFs. Dural AVFs account for 10–15% of all intracranial malformations 11 and the CT findings are highly variable and often non-specific. The lack of temporal resolution and peripheral location of dural feeders makes CTA identification difficult. Signs may be subtle in low-grade lesions where cortical venous reflux or venous ectasia are absent (16).

Given these findings, our study showed higher sensitivity and specificity for CTA lobar than other studies (10).

The high-diagnostic value of new CT scanners and protocols raises the question of whether DSA should be considered the gold standard. Rather, it should be clarified how to select the 2–3% of patients with lobar ICH in whom CTA fails in diagnosis.

Here, as in many other areas, deep learning can help not only in the pure detection of bleeding but also in the immediate assignment of a possible cause of bleeding(17–23).

It is important to close the diagnostic gap of CTA, and DSA should be dispensed in the future. Recent studies have demonstrated an improvement in the diagnostic accuracy of CTA (3D-CTA and 4D-CTA) in detecting underlying vascular abnormalities (24–27). The improvement in 4D-CTA combines traditional 3D-CTA with the 4th dimension of time. 4D-CTA has the potential to exclude the vascular causes of hematoma in many cases, thereby avoiding unnecessary DSA investigations. However, the lack of relevant studies and the small number of patients included in the four studies reviewed has highlighted that it would be advantageous to perform further studies comparing 4D-CTA with DSA in larger prospective patient cohorts to increase the evidence base that would inform clinical decision-making in patients with spontaneous ICH (28). CAA is an important reason for lobar ICH, especially in elderly patients, which cannot be verified with DSA and CTA but with MRI (5, 6, 29). After non-contrast CT, CTA is the next, easiest, and most important choice for detecting the vascular causes of lobar ICB. Non-contrast CT alone cannot predict the presence or absence of an underlying vascular lesion (77% sensitivity and 84% specificity) (30).

The CTA should be performed acutely in all patients, except those at low risk of having an underlying macrovascular cause (10).

The DSA has an advantage over CTA in terms of time resolution, which characterizes the direction and rate of blood flow within the cranial circulation. This allows for the detection of arteriovenous shunting within a dural arteriovenous fistula that might otherwise be missed on CTA.

Elmegiri et al. demonstrated that MRI had a higher sensitivity for detecting structural and local causes than CT and angiography, whereas angiographic imaging was highly specific for structural abnormalities (31).

Identification of small vessel disease (moderately severe leukoaraiosis or lacunar infarction on CT), in combination with abnormal CTA and pre-ICH hypertension ICH, can predict which patients have a low yield of an intracranial macrovascular cause (32, 33).

One major limitation is the lack to include patients with non-lobar ICH location. This happened with the experience of clinical routine that most central/deep ICH is caused by arterial hypertension.

In summary, CTA has almost the same diagnostic capabilities as DSA; therefore, diagnostic tools or scoring systems should be developed to select a small number of patients whose vascular pathology is not detected by CTA, but by DSA (14).

## Data availability statement

The raw data supporting the conclusions of this article will be made available by the authors, without undue reservation.

## Ethics statement

The studies involving human participants were reviewed and approved by University Hospital Essen. The patients/participants provided their written informed consent to participate in this study.

## Author contributions

J-CA, SF, JE, SS, and CD contributed to conception and design of the study. CD organized the database. J-CA and HN performed the statistical analysis. J-CA wrote the first draft of the manuscript. DP, KW, YL, BF, ND, CW, MK, MF, and CD wrote sections of the manuscript. All authors contributed to manuscript revision, read, and approved the submitted version.

## Conflict of interest

The authors declare that the research was conducted in the absence of any commercial or financial relationships that could be construed as a potential conflict of interest.

## Publisher's note

All claims expressed in this article are solely those of the authors and do not necessarily represent those of their affiliated organizations, or those of the publisher, the editors and the reviewers. Any product that may be evaluated in this article, or claim that may be made by its manufacturer, is not guaranteed or endorsed by the publisher.
